# Systems analysis of subjects acutely infected with the Chikungunya virus

**DOI:** 10.1371/journal.ppat.1007880

**Published:** 2019-06-18

**Authors:** Alessandra Soares-Schanoski, Natália Baptista Cruz, Luíza Antunes de Castro-Jorge, Renan Villanova Homem de Carvalho, Cliomar Alves dos Santos, Nancy da Rós, Úrsula Oliveira, Danuza Duarte Costa, Cecília Luíza Simões dos Santos, Marielton dos Passos Cunha, Maria Leonor Sarno Oliveira, Juliana Cardoso Alves, Regina Adalva de Lucena Couto Océa, Danielle Rodrigues Ribeiro, André Nicolau Aquime Gonçalves, Patricia Gonzalez-Dias, Andreas Suhrbier, Paolo Marinho de Andrade Zanotto, Inácio Junqueira de Azevedo, Dario S. Zamboni, Roque Pacheco Almeida, Paulo Lee Ho, Jorge Kalil, Milton Yutaka Nishiyama, Helder I. Nakaya

**Affiliations:** 1 Bacteriology Laboratory, Butantan Institute, São Paulo, Brazil; 2 Department of Clinical and Toxicological Analyses, School of Pharmaceutical Sciences, University of São Paulo, São Paulo, Brazil; 3 Departamento de Biologia Celular, Molecular e Bioagentes Patogênicos, Faculdade de Medicina de Ribeirão Preto, University of São Paulo, Ribeirão Preto, Brazil; 4 Health Foundation Parreiras Horta, Central Laboratory of Public Health (LACEN/SE), State Secretary for Health, Sergipe, Brazil; 5 Special Laboratory for Applied Toxinology, Butantan Institute, São Paulo, Brazil; 6 Respiratory Diseases Division, Virology Center, Adolfo Lutz Institute, Sao Paulo, Brazil; 7 Laboratory of Molecular Evolution and Bioinformatics, Department of Microbiology, Biomedical Sciences Institute, University of São Paulo, São Paulo, Brazil; 8 Division of Immunology and Molecular Biology Laboratory, University Hospital/EBSERH, Federal University of Sergipe, Sergipe, Brazil; 9 QIMR Berghofer Medical Research Institute, Brisbane, Queensland, Australia; 10 Bacteriology Service, Bioindustrial Division, Butantan Institute, São Paulo, Brazil; 11 Heart Institute, Faculty of Medicine, University of São Paulo, São Paulo, Brazil; University of Wisconsin, UNITED STATES

## Abstract

The largest ever recorded epidemic of the Chikungunya virus (CHIKV) broke out in 2004 and affected four continents. Acute symptomatic infections are typically associated with the onset of fever and often debilitating polyarthralgia/polyarthritis. In this study, a systems biology approach was adopted to analyze the blood transcriptomes of adults acutely infected with the CHIKV. Gene signatures that were associated with viral RNA levels and the onset of symptoms were identified. Among these genes, the putative role of the Eukaryotic Initiation Factor (eIF) family genes and apolipoprotein B mRNA editing catalytic polypeptide-like (APOBEC3A) in the CHIKV replication process were displayed. We further compared these signatures with signatures induced by the Dengue virus infection and rheumatoid arthritis. Finally, we demonstrated that the CHIKV *in vitro* infection of murine bone marrow-derived macrophages induced IL-1 beta production in a mechanism that is significantly dependent on the inflammasome NLRP3 activation. The observations provided valuable insights into virus-host interactions during the acute phase and can be instrumental in the investigation of new and effective therapeutic interventions.

## Author summary

The Chikungunya virus (CHIKV) has infected millions of people worldwide and presents a serious public health issue. Acute symptomatic infections caused by contracting this mosquito-transmitted arbovirus are typically associated with an abrupt onset of fever and often debilitating polyarthralgia/ polyarthritis, as well as prolonged periods of disability in some patients. These dramatic effects call for a careful evaluation of the molecular mechanisms involved in this puzzling infection. By analyzing the blood transcriptome of adults acutely infected with CHIKV, we were able to provide a detailed picture of the early molecular events induced by the infection. Additionally, the systems biology approach revealed genes that can be investigated extensively as probable therapeutic targets for the disease.

## Introduction

The Chikungunya virus (CHIKV) is a mosquito-borne reemerging arbovirus responsible for intermittent and devastating outbreaks [1]. The largest epidemic of CHIKV ever recorded started in Africa in 2004 and has spread globally, reaching the Americas in 2014. The disease has afflicted four continents, affected more than 100 countries and infected over 10 million people [2, 3]. Its global impact is still growing [4]. CHIKV has spread rapidly through several Brazilian states and infected a total of 20,598 individuals in 2015 [5]; furthermore, more than 200,000 suspected cases were reported in 2017–2018 [6].

The CHIKV acute infection typically results viremia for 5–7 days. The symptoms are characterized by fever, rash and severe polyarthralgia/polyarthritis that can become chronic and persist from months to years [7]. Mortality rates are estimated to be approximately 0.1% [8]; however, the high attack rates (up to 30–75%) can result in considerable economic burden [9, 10]. The current mainstay of treatment is the use of NSIADs and/or acetaminophen, although relief is often inadequate and more effective treatments are actively being sought [11]. Several studies have sought to understand the CHIKV disease pathogenesis and virus-host interactions better [12] using *in vitro* approaches [13] or animal models [14, 15].

Systems biology approaches have been successfully applied to identify molecular signatures associated with infections [16] and vaccination [17]. In this study, a systems approach was performed to investigate subjects who were naturally infected with CHIKV during the 2016 epidemics in the state of Sergipe, Brazil. The blood transcriptome analyses performed revealed key genes and pathways involved in acute CHIKV infection, thereby providing important insights into how CHIKV interacts with the host’s immune system. These analyses helped uncover potential drug targets for improving CHIKV therapy.

## Results

### Clinical information and diagnosis of CHIKV patients and controls

**Fig 1:**
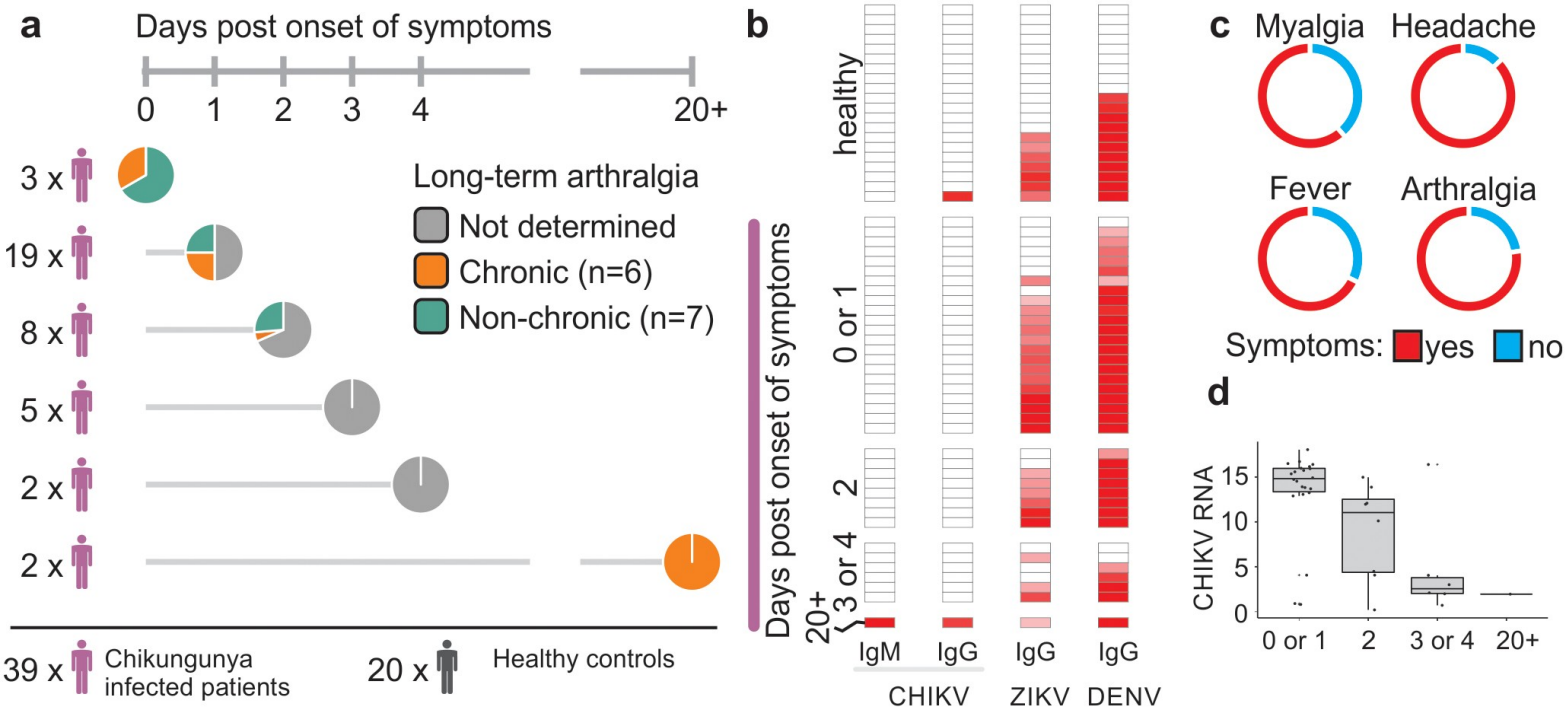
CHIKV sample collection, clinical data and diagnoses. (a) Study design representing the number of individuals and the number of days from the onset of symptoms and till blood sample collection. The status of long-term arthralgia was assessed in some of the patients and indicated by the following color scheme: chronic (orange), non-chronic (green) and undetermined (gray). (b) Serodiagnoses for CHIKV, DENV and ZIKV showing highest immunity for Dengue and Zika in the red scale (n = 37). The number of days post onset of symptoms are shown for all CHIKV patients (purple vertical bar) (c) Doughnut chart indicating the most prevalent symptoms reported by CHIKV patients. (d) CHIKV RNA (inverse Ct) in the subgroups determined by the number of days between the onset of symptoms and the collection of blood samples (n = 37).

In an endemic area for the Chikungunya, Zika and Dengue viruses [18] in the northeast of Brazil, whole blood samples were collected from 39 adult patients with symptoms consistent with arboviral infection, including fever, arthralgia, headache and/or muscle pain. Most patients (n = 22) reported that the onset of the symptoms had occurred on the same day or one day prior to the blood collection (Fig 1a and S1 Table). Eight patients reported having had symptoms for two days, and seven reported symptoms surfacing three to four days prior to the blood collection. Two patients reported pain for the past 20 days or more (Fig 1a). The presence of CHIKV RNA (as determined by real-time RT-PCR) or CHIKV-specific IgM (as determined by serodiagnostic ELISA) was confirmed in serum samples of all except five patients (S1 Table). These five patients had borderline levels of CHIKV RNA (n = 4) or had no serum available (n = 1) and were included in the study based on their clinical symptoms (S1 Table). All samples were negative for Zika and Dengue RNA (S1 Table). The most frequent symptoms in CHIKV-infected patients were fever, arthralgia, headache and myalgia (Fig 1c). As expected [16], the highest levels of CHIKV RNA were found in serum samples from patients in whom the onset of the disease was recent (Fig 1d).

Blood was also collected from 20 healthy subjects from endemic and non-endemic regions in Brazil. None of the subjects showed symptoms of viral infection, and they tested negative for CHIKV, Zika and Dengue RNA. Serodiagnostic ELISAs were also performed to detect the presence of IgG antibodies specific to the Zika and/or Dengue viruses (Fig 1b, S1 Table). IgG antibodies specific to CHIKV were detected in only one subject.

### The impact of CHIKV acute infection on peripheral blood transcriptome

The peripheral blood transcriptomes of CHIKV patients were compared with those from healthy controls to understand the transcriptional changes associated with acute CHIKV infections. Although the CHIKV RNA levels had a small contribution to the variance observed in the transcriptomes, individuals were not grouped by the days post the onset of symptoms or by their infection status (i.e., healthy individuals and CHIKV patients) (S1b Fig).

**Fig 2:**
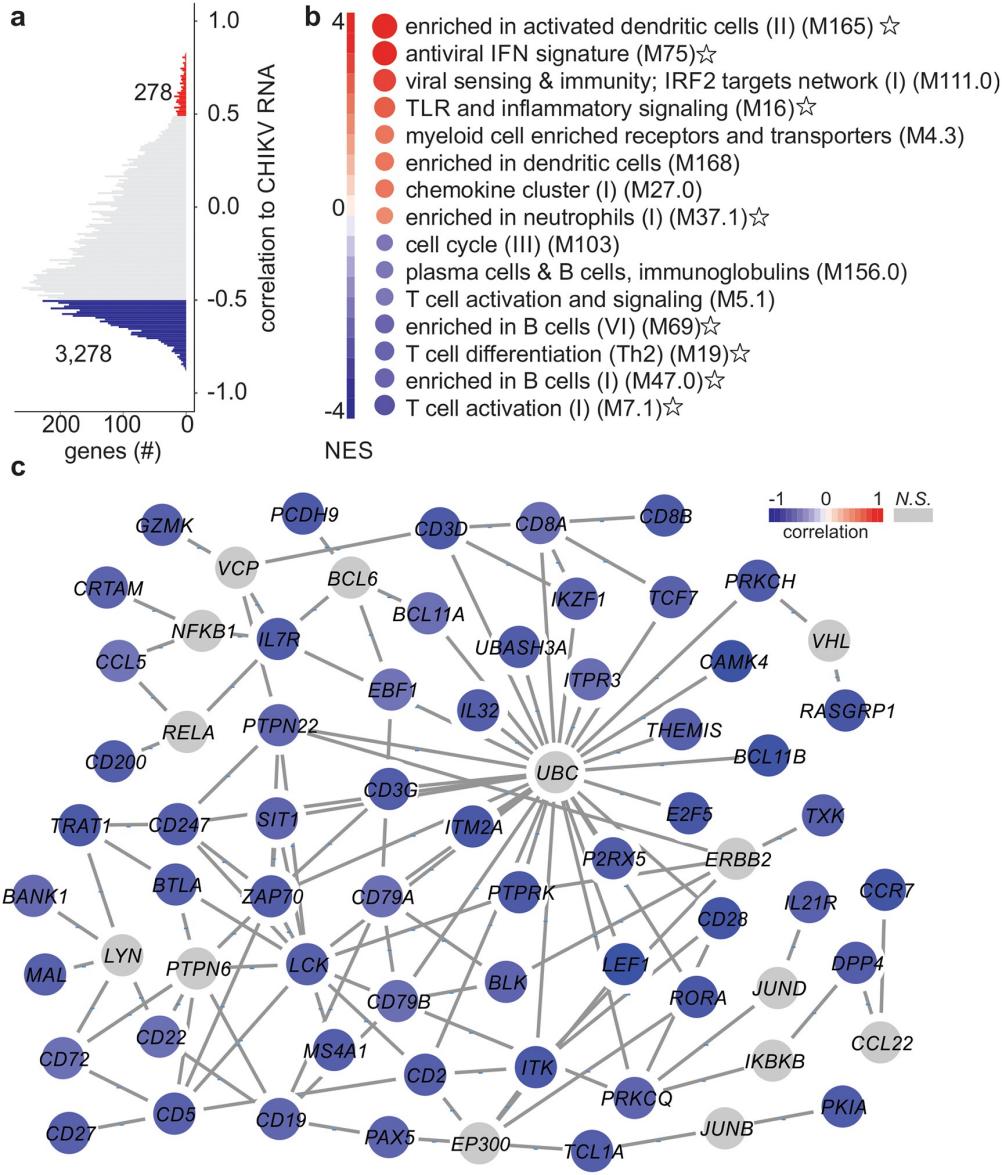
Gene signatures associated with levels of CHIKV RNA. (a) Representation of genes whose expression were correlated (Pearson’s r) with the levels of CHIKV RNA (inverse Ct). The respective number of genes positively (red) or negatively (blue) correlated with the levels of CHIKV RNA are shown (p-adjusted value < 0.01 and |R| > 0.5). (b) Results of GSEA conducted using the correlation values between expression and inverse Ct as ranks and BTMs as gene sets. The stars correspond with the gene sets used to construct the networks shown in panel C (negatively correlated pathways) and S2 Fig (positively correlated pathways). (c) Minimum network constructed using the gene sets that presented a negative NES score with the NetworkAnalyst tool. The protein-protein interaction (PPI) data was based on InnateDB and the graph was generated with the Cytoscape tool. Blue nodes represent genes that are negatively correlated with levels of CHIKV RNA and the color scheme indicates its strength. The gray nodes were added by NetworkAnalyst and are not a part of the correlated genes.

Correlation analysis of gene expression and the levels of CHIKV RNA in the peripheral blood of the infected individuals revealed approximately 3,500 genes associated with CHIKV infection. The expression of most of these genes (> 85%) was negatively correlated with CHIKV RNA levels (Fig 2a). Using the correlation values as rank and Blood Transcription Modules (BTM) as gene sets, we ran a gene set enrichment analysis (GSEA) to reveal the BTMs related to the levels of CHIKV RNA. BTMs associated with innate immune cells (dendritic cells and neutrophils), antiviral response, inflammation and toll-like receptor signaling (Fig 2b and S4 Fig) were positively associated with the level of CHIKV RNA. BTMs negatively associated with the level of CHIKV RNA (i.e., with a negative normalized enrichment score, NES) were related to adaptive immune response, such as cell proliferation, activation and differentiation of B and T lymphocytes, as well as chemokines and cytokines that could be due to the CHIKV infection-related lymphopenia (Fig 2b) [19]. A gene network was subsequently constructed by connecting the genes related to BTMs with a negative NES score (Fig 2b) by utilizing the protein-protein interaction (PPI) data from InnateDB. Genes related to adaptive immune response and cell recruitment such as *CCL5*, *CD8A* and *CD8B*, *CD3D*, *CD19*, *CCR7* and *TCF7*, among others, appeared in the network (Fig 2c). Several of these genes are related to the homing/recruiting of the effector/memory T lymphocytes as well as the activation and induction of memory cells. The same process was performed for BTMs with positive NES scores (S2 Fig). Almost all the genes in this network were human type I ISGs (as defined by Interferome V2), which is entirely consistent with the stimulation of the type I IFN response by CHIKV dsRNA [20]. IRF7 is a central highly inducible transcription factor for driving the type I IFN responses to CHIKV [21], and OAS3, IFIT1, MX2 and RSAD2 (viperin) are key antiviral effectors [22]. SERPING1 (C1 inhibitor) is a novel finding since this inhibitor of the classical pathway of the complement system has not been associated with arthritogenicalphaviruses [23], although it is up-regulated in monocytes in HIV infections [24].

**Fig 3:**
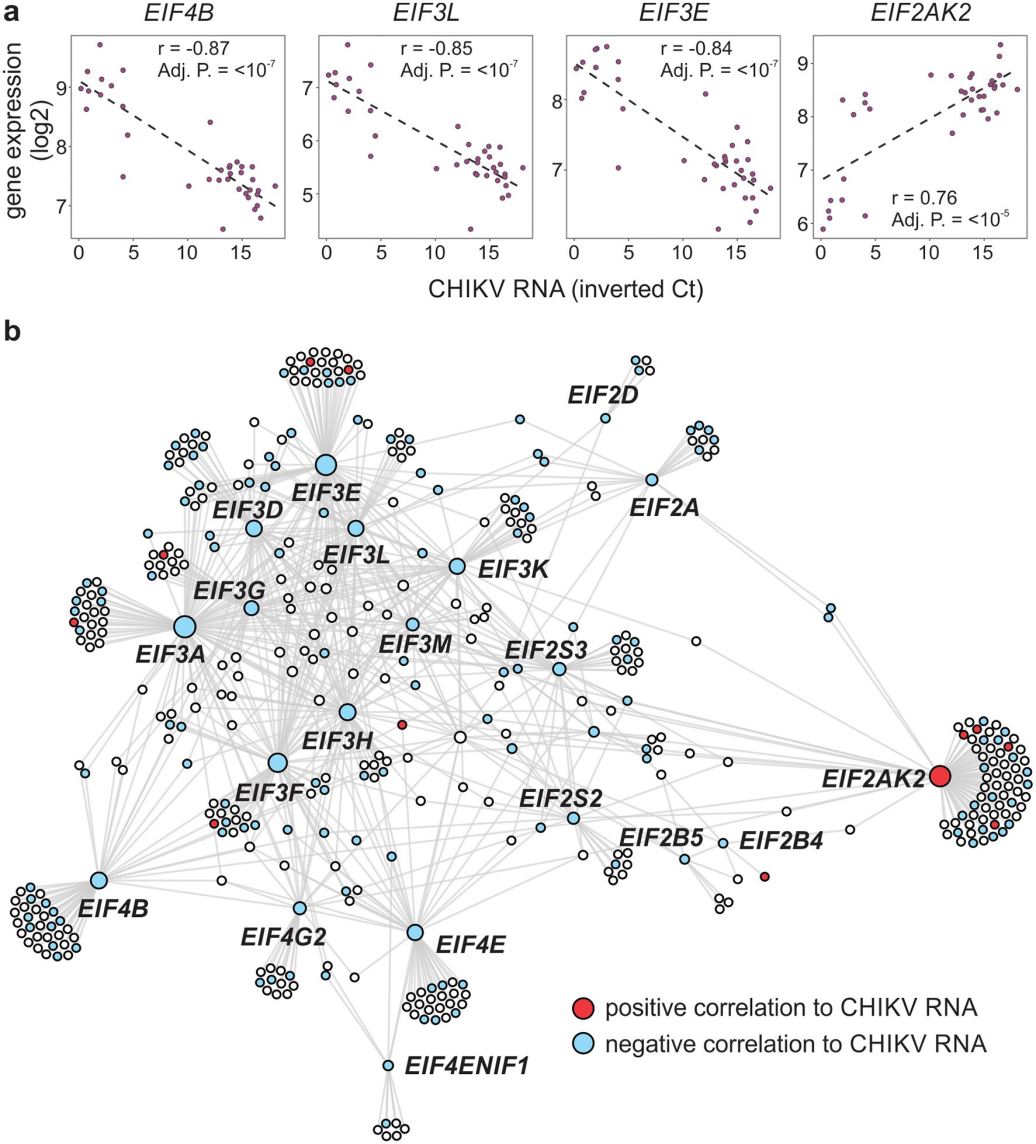
Eukaryotic Initiation Factor (eIF) genes associated with CHIKV RNA levels. (a) Representation of the negative correlation of *eIF* genes with CHIKV RNA levels (inverse of Ct). (b) Network constructed with *eIF* genes correlating with CHIKV RNA levels and integrated with PPI data. The NetworkAnalyst tool (InnateDB PPIs; First Order) was used to construct the network and the Gephi program was used for visualization. The color of the nodes illustrates whether the correlation between gene expression and CHIKV RNA levels is positive (red) or negative (blue).

The expression of several eukaryotic translation initiation factors (eIFs) was negatively correlated with the level of CHIKV RNA (Fig 3), indicating that these genes can play a major role in viral replication. The eIFs are important proteins for controlling the translation initiation processes involved in host and viral protein syntheses [25]. A network with eIF genes was constructed (Fig 3b) among which *EIF4B*, *EIF3L*, *EIF3E* and *EIF2AK2* presented the highest negative correlation with levels of CHIKV RNA (Fig 3a). Only the *EIF2AK2* gene (also known as PKR) had a positive correlation with CHIKV RNA levels. This protein is activated by alphavirus infections and contributes to the characteristic shut-down of host cell protein synthesis, thereby allowing the preferential translation of capped viral RNA [26].

### Consistent transcriptome changes during CHIKV infection

We compared the expression profiles between all CHIKV-infected patients and healthy controls and identified 3,059 up- and 1,898 down-regulated genes in the CHIKV patients. The script and data to reproduce this result are available at https://github.com/csbl-usp/csbl_chikungunya_edgeR.

**Fig 4:**
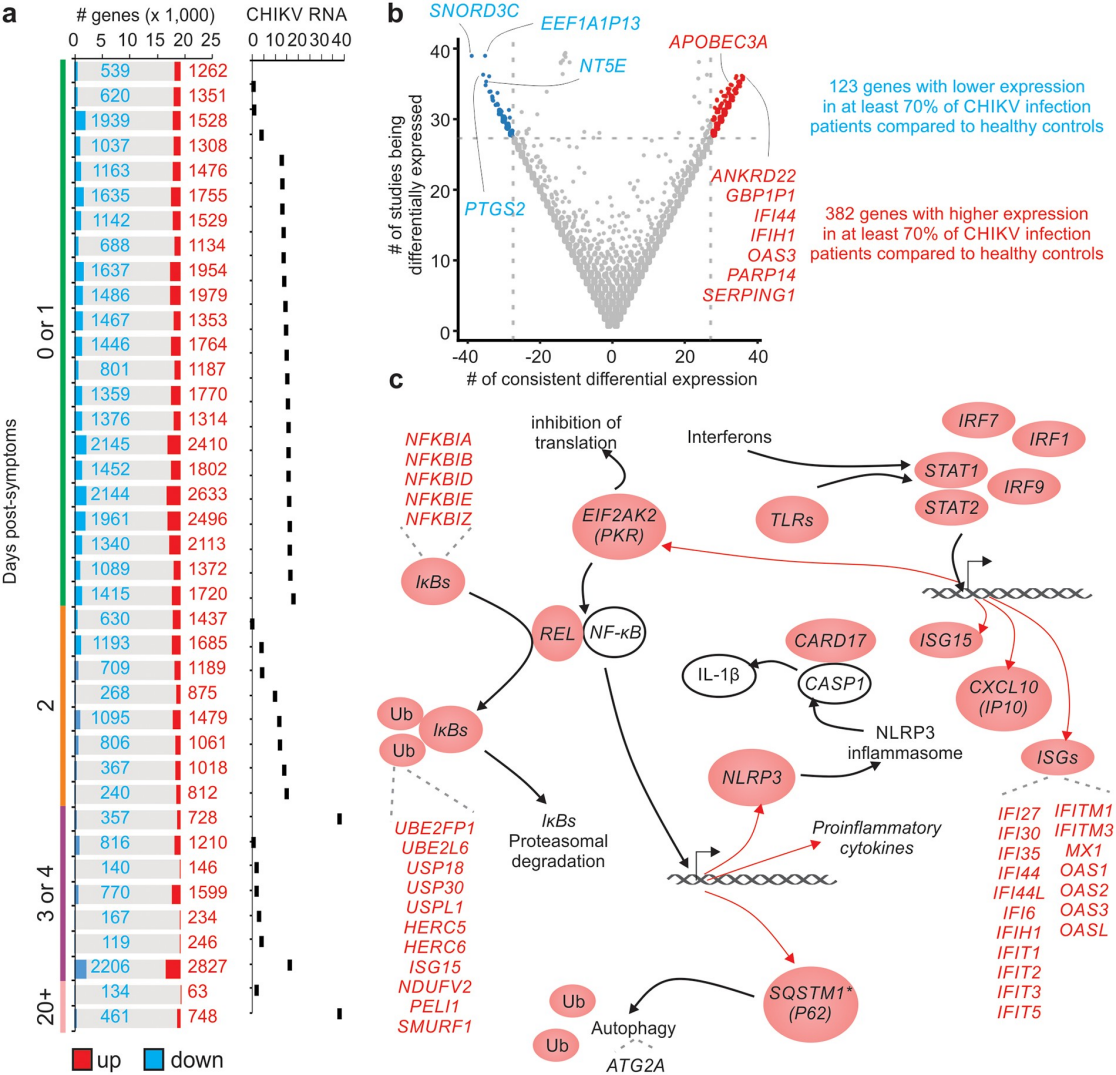
Differentially expressed genes in CHIKV patients. (a) The profile of each infected patient was compared with all of the healthy controls to indicate the number of up- and down-regulated genes. The colors represent the number of up-regulated (red) or down-regulated (blue) genes and the respective number of genes in each group. The bar graph to the right indicates the CHIKV RNA levels for each patient. (b) The meta-volcano analysis showed genes that were consistently differentially expressed (log2 fold change > 1 and adjusted p-value < 0.05) in at least 70% of the comparisons from CHIKV patients versus healthy controls. The x-axis represents the number of consistent differential expressions of each gene while the y-axis represents the number of studies in which the genes were classified as a DEG. (c) Graphical representation of the putative relationship between some of the genes that were classified as consistently up-regulated in (b).

Due to the natural heterogeneity in human cohorts, we also performed differential expression analyses between each infected patient and the group of healthy controls. The patients whose blood samples were collected in the initial days post the onset of symptoms revealed higher numbers of DEGs (differentially expressed genes) detected by RNA-seq analysis and higher mean CHIKV RNA levels (Fig 4a). The DEG list for each patient was subsequently combined into a meta-volcano plot (Fig 4b). This plot displays the number of genes whose expression was consistently altered in most patients. Approximately 70% of the CHIKV-infected patients showed down-regulation of 123 genes and up-regulation of 382 genes (Fig 4b). *APOBEC3A*, *IFI44* [15] and *OAS3* were the most represented up-regulated genes and *NT5E* (also known as CD73), *PTGS2*, *SNORD3C*, and *EEF1A1P13* were the most represented down-regulated genes among the CHIKV patients (Fig 4b). APOBEC3 family members are important cytidine deaminases that control *inter alia* HIV replication [27] but have, to the best of our knowledge, not previously been associated with alphaviral infections. Interestingly, the analysis also identified *NT5E*/CD73 (Fig 4b) that is an ecto-5′-nucleotidase that has been described as an important molecule for recovery of the endothelial barrier after Dengue 2 infection [28]. Vascular leakage and shock have also been reported as a severe manifestation of CHIKV [29].

Through literature curation of the CHIKV gene signature, we created a network that provides novel insights into CHIKV immunobiology (Fig 4c). CHIKV infection could induce the expression of several ISGs, as well as the activation of TLRs, EIF2AK2 and IkBs. Up-regulation of *STAT1*, *STAT2*, *IRF9*, *IRF1* and *IRF7* can induce the expression of ISGs, which are important antiviral effectors. Interestingly, the CHIKV infection led to the up-regulation of several proinflammatory genes such as IκBs-related genes that are important to NFκB activation and proinflammatory cytokines production when phosphorylated. It is integral to mention here that we also detected the up-regulation of *REL*, which is a subunit of NFκB.

DEGs related to the NLRP3 inflammasome were also detected. Chemotherapeutic inhibition of the NLRP3 inflammasome was recently shown to inhibit CHIKV arthropathy in a mouse model [30]; the current study, therefore, confirms this pathway as a potential drug target in humans. *Nlrp3* is transcriptionally regulated to guarantee high protein levels for the activation of the NLRP3 inflammasome. Its activation could lead to autophagy (*SQSTM1*) [31], apoptosis of the infected cells and the production of other cytokines (*CARD17*) [32] in response to the infection (Fig 4c). Taken together, our results expand the knowledge available about host-CHIKV interaction and provide potential therapeutic strategies that target the NLRP3 inflammasome [32].

The random forest method, a machine learning approach, was also employed to rank the importance of the 505 DEGs mentioned in the meta-volcano analysis (Fig 4b) in predicting the CHIKV infection status. S3 Fig displays the 38 genes that better distinguish between the infected patients and healthy control samples from 100 feature selection interactions (S3a Fig). On each interaction, the 59 infected patients and healthy control samples were split into training (70%) and testing (30%) sets. We subsequently applied different machine learning models (CART, kNN, SVM, RF, xgBoost, bayesglm, adaboost, ada, rpart, nnet, plr, regLogistic and dnn) on the training set to predict the testing set. Among all the models, the RF (Random Forest) model displayed better performance (S3b Fig) with an accuracy of 94.12% (Confident Interval: 71.31% up to 99.85%).

SerpinG1 is the C1 complement inhibitor (inhibiting the classical pathway of the complement) but also inhibits other proteases including MASP-1 and MASP-2 in the lectin pathway. Circulating SerpinG1 protein levels increase ~2.5 folds during inflammation [33]. Furthermore, during HIV infections, SerpinG1 mRNA levels increased in the circulating monocytes [24]. The lectin pathway has been implicated as causing the tissue damage during alphaviral arthritis caused by the Ross River virus [23], and SerpinG1 upregulation may act to limit such damage [34].

We next performed single sample GSEA analysis using the log2 fold-change values of each patient compared to the group of healthy controls as ranks and the BTMs as gene sets (S4 Fig). Similar to the results presented in Fig 2, we observed that the up-regulated BTMs were related to innate immunity and antiviral responses involving dendritic cells and monocytes activation. This is highly consistent with the work of Michlmayr et al [35].

### Potential signatures of CHIKV-induced chronic arthralgia

The patients (n = 13) who agreed to return for clinical follow-up examinations were split into those with chronic and those with non-chronic arthralgia (see Methods). These two groups showed no significant difference in the mean levels of CHIKV RNA in the serum (S5a Fig). Compared to the healthy controls, a total of 1,262 and 1,862 genes were consistently differentially expressed in most of the chronic and non-chronic patients respectively (S5b Fig). Of them, 514 genes were commonly up-regulated and 337 were down-regulated in both groups (S5c Fig). Additionally, a high positive correlation was observed between the mean log2 fold change of chronic and non-chronic patients when compared to healthy controls (S5d Fig). However, few genes presented an expression profile that differed between chronic and non-chronic patients (S5d Fig). *HLA-DRB5* was up-regulated in most non-chronic patients and down-regulated in most chronic patients (S5d Fig). However, such changes may merely be associated with the diminishing of the acute adaptive T cell responses [22]. Of considerably greater interest are the genes that are up-regulated in most chronic patients relative to non-chronic patients. The *EIF1AY* gene belongs to the family of eukaryotic translation initiation factors (and are displayed in Fig 3). Interestingly, the *DDX3Y* is an RNA helicase that was described as an important effector of the herpes virus replication and propagation [36] but has not been described in the context of the CHIKV. However, our analyses might simply be a reflection of a greater number of males in the chronic group since several of the genes identified were Y linked. This analysis, thus, provides no support for the notion of the onset of new pathological processes in chronic diseases—an observation in agreement with murine studies [22].

### Modular expression analysis of CHIKV infection

**Fig 5:**
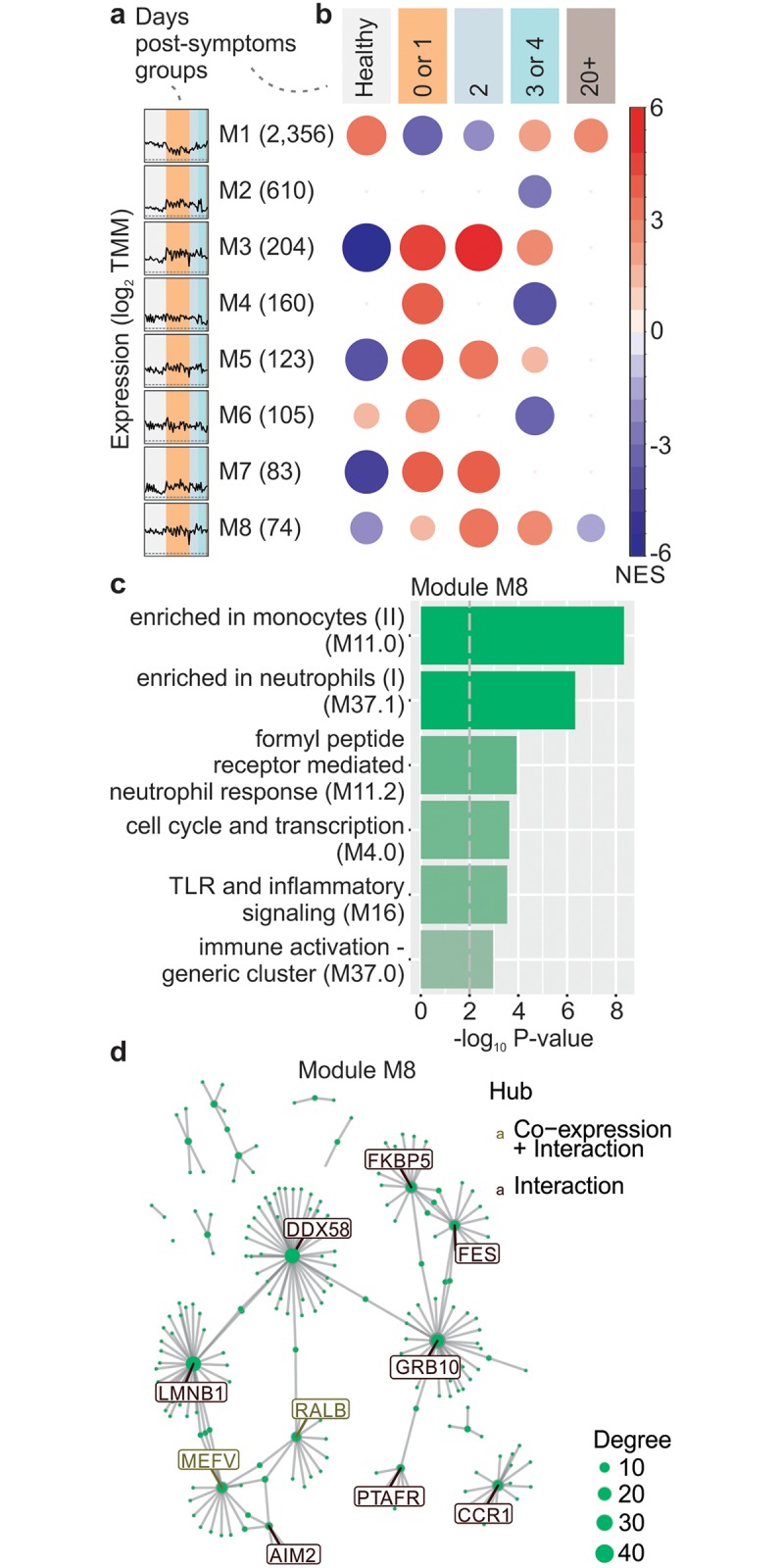
Modular analysis of CHIKV infection. (a) Expression profile of all the eight modules obtained through the CEMiTool analysis. The colors of rectangles represent the days post the onset of symptoms, and the black line represents the mean expression of all the genes inside the module. (b) Results of the gene set enrichment analysis showing the module activity for each subgroup. The size and color of the circles represent the normalized enrichment score (NES). (c) Over-representation analysis of module M8 using BTM pathways as gene sets. The pathways were ordered by significance as indicated on the x-axis. (d) Gene network of module M8 for the top ten most connected genes (hubs) represented as nodes and their protein-protein and co-expression interactions as edges. The size of the node represents its degree of connectivity.

We performed a gene co-expression network analysis using the expression profiles of all the patients and healthy controls. CEMiTool [37] identified eight co-expression modules containing 74 to over 2,000 genes (Fig 5a). The expression activity of some of these modules were altered in healthy controls or in patients that had different days post the onset of symptoms (Fig 5b). Module M8, which shows higher activity in patients with two to four days of symptoms was enriched for monocytes and neutrophils (Fig 5c). A monocyte centric response in peripheral blood from pediatric CHIKV patients has previously been documented [35] and a protective role was observed in murine models [22]. Neutrophil responses have hitherto not been described for alphaviral infections, with granulocytosis in non-human primates being predominantly unremarkable [38] and a pathogenic role for neutrophils only evident in CCR2^-/-^ mice [39]. Nevertheless, although previously thought to characterize bacterial infections, neutrophil responses are now recognized in a range of acute viral infections [40, 41].

In module M8, one of the hubs contained the gene *DDX58* (also known as *RIG-1*). RIG-I is well described as a dsRNA sensor for CHIKV [42] and is instrumental for driving the protective and pathogenic type I IFN responses [20].

Another gene identified is *AIM2* (Fig 5d), an interferon-inducible cytoplasmic dsDNA sensor that activates the inflammasome and triggers pyroptosis. The role of AIM2 and self-DNA in driving polyarthritis has been proposed [43], with CHIKV infection-mediated cytopathic effects conceivably providing the DNA to stimulate this pathway.

### Comparing CHIKV signature with rheumatoid arthritis and Dengue infection signatures

**Fig 6:**
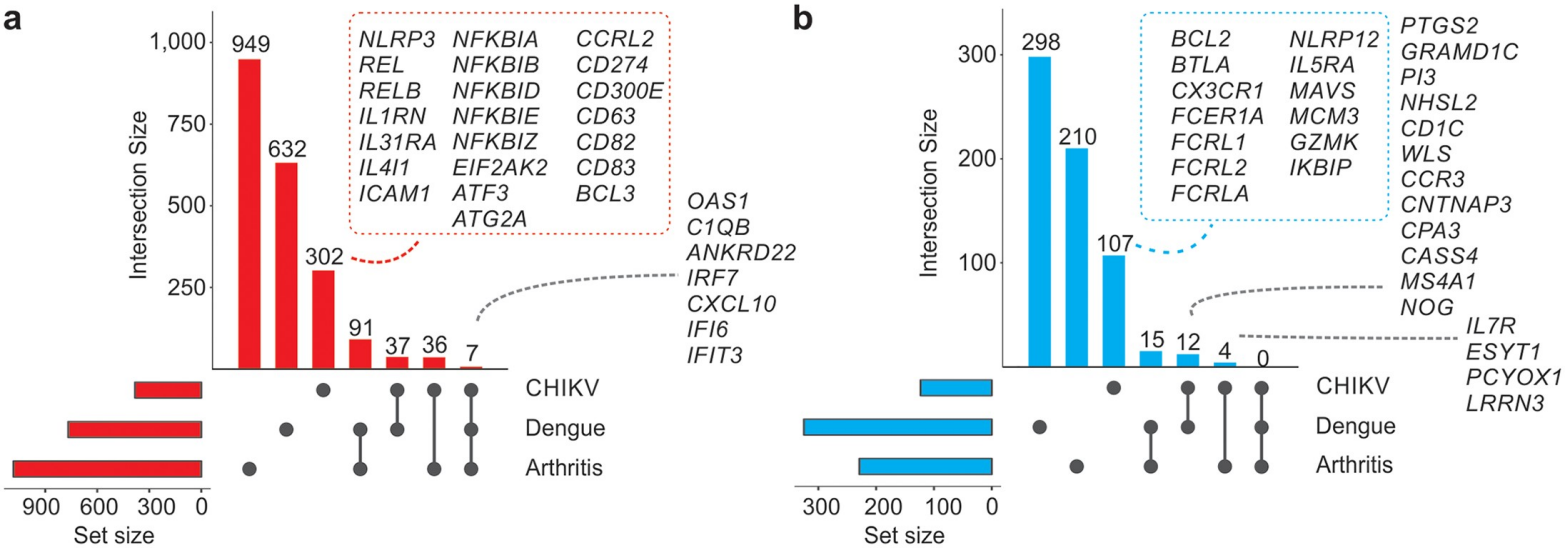
Comparing CHIKV signatures with those from rheumatoid arthritis and Dengue infection. (a) Genes up-regulated in patients infected with either CHIKV or Dengue or rheumatoid arthritis (RA) patients compared to their respective controls. The size of the bar represents the quantity of up-regulated genes detected in one or more groups (indicated as a gray circle in the graph below). Representative up-regulated genes are displayed. The gray circles below the bars indicate the groups that share the same up-regulated genes. (b) The same approach as in (a) but for the down-regulated genes.

The CHIKV signature was compared with signatures from another viral infection (Dengue virus) and from rheumatoid arthritis (RA) to check the extent to which the gene signature was specific to CHIKV infection. We re-analyzed two publicly available blood transcriptome datasets and identified the genes whose expression was altered in the Dengue infected patients and RA patients compared to healthy controls. A total of 949, 632 and 302 genes were identified as being up-regulated only in RA, Dengue infection and CHIKV infection, respectively (Fig 6a). Among those, seven up-regulated genes were shared by all the three signatures, including the following: *OAS1*, *C1QB*, *ANKRD22*, *IRF7*, *CXCL10*, *IFI6* and *IFIT3* (Fig 6a); all of these are interferon-inducible genes. More than 300 genes were exclusively up-regulated in CHIKV infection, including the inflammasome-related *NLRP3* genes, NFκB-related genes and genes that belong to the Th2 response (*ILI31RA*, *IL4I1*) [44] (Fig 6a).

Although no gene was down-regulated in all the three conditions, genes such as *IL7R*, *ESYT1*, *PCYOX1* and *LRRN3* were commonly down-regulated in CHIKV infection and RA samples (Fig 6b). The cytokine IL-7 is crucial for the survival of naïve and memory T cells, which are important effectors against several pathogens including viral infections. The receptor IL-7R is expressed on the surface of these cells and was shown in an animal model to prevent the chronicity induced by lymphocytic choriomeningitis virus due to the enhancement of CD8 T cells’ response and prevention of its exhaustion [45]. We identified a specific CHIKV signature composed of 107 down-regulated genes that includes *CX3CR1*, *BTLA*, *BCL2*, *GZMK* and three genes that encode Fc receptor-like glycoproteins (Fig 6b) that could be related to the lymphopenia induced by CHIKV infection [38]. The down-regulation of CX3C chemokine receptor 1 (*CX3CR1*) seems to be in agreement with the results of this study that showed that several T cell effector/differentiation genes were down-regulated in CHIKV infection. CX3CR1 has been considered as an important marker of CD8 memory cells [46]. Moreover, *BCL-2* down-regulation could be a mechanism of CHIKV host response to induce apoptosis of infected cells.

### CHIKV activates the inflammasome *in vitro*

**Fig 7:**
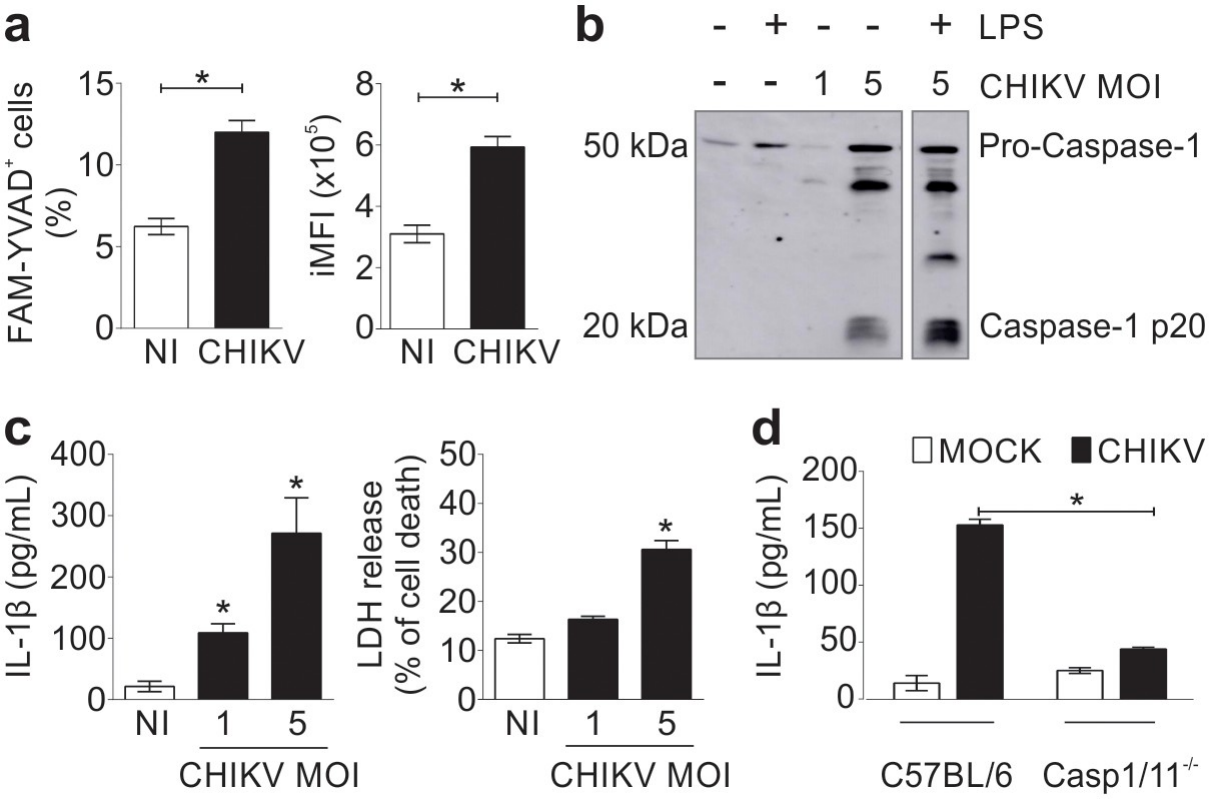
CHIKV activates inflammasome in murine macrophages. (a) Mouse bone marrow-derived macrophages (BMDMs) were infected with CHIKV with a PFU/cell of 5 or not infected (NI); after 24 hours of infection, the cells were stained for active caspase-1 with FAM-YVAD. The percentage (left) and the integrated mean of fluorescence (iMFI) of the activated cells (right) is shown. (b) After 24 hours of infection, supernatants were harvested from non-infected (NI) or CHIKV-infected BMDMs treated with LPS as depicted and levels of cleaved caspase-1 (p20) were detected by western blotting in the supernatant. (c) LPS-primed BMDMs were infected with CHIKV with a PFU/cell of one or five. After 24 hours of infection, the levels of IL-1β (left) and LDH (right) in the cell-free supernatants were measured. (d) Production of IL-1β by BMDMs from wild type (C57BL/6) or Casp1/11 deficient mice 24 hours post-CHIKV infection. Data from (a), (c) and (d) are represented as the mean ± SD of the triplicate samples and are representative of the three independent experiments that yielded similar results. Statistical analysis was performed with Student’s t-test. Asterisks indicate statistically significant differences. *P < 0.05.

Since the inflammasome-related genes are exclusively up-regulated in CHIKV infection and were described before in this context [30], we decided to provide a deeper proof-of-concept related to these findings. The murine bone marrow-derived macrophages were infected with CHIKV virus and the readouts of the inflammasome activation were measured. A specific dye was used that binds to active caspase-1 (FAM-YVAD) to assess caspase-1 activation with CHIKV-induced caspase-1 activation measured as a percentage of FAM-YVAD+ cells as well as the integrated mean of fluorescence (iMFI) (Fig 7a). Caspase-1 activation was further assessed by western blot and detected caspase-1 cleavage as indicated by the presence of Casp1 p20 in cells infected with PFU/cell of 5 (Fig 6c). CHIKV infection at PFU/cell of 1 and 5 induced IL-1β production and LDH release [indicative of pyroptosis [47]] (Fig 6d). IL-1β production was dependent on inflammasome activation since Casp1/11 deficient macrophages failed to trigger IL-1β production in response to the infection (Fig 6e). These data support the view that the inflammasome is activated in response to CHIKV infection and are consistent with our transcriptional analyses [and murine studies [30]].

## Discussion

CHIKV re-emergence in the past 15 years has led to major epidemic outbreaks in Asia, Africa, the Indian Ocean and more recently in the Americas after decades of intermittent outbreaks [48]. Despite considerable progress in understanding the infection, much of the host-pathogen interplay remains obscure.

Systems biological approaches can provide comprehensive and unbiased dissections of the complex interactions between genes and proteins in infections [16]. Relevant studies related to the natural infection by CHIKV were recently published. However, these were restricted to the analysis of a limited number of proteins [49] or of children infected with the virus [50]. Since it is extremely difficult to acquire sufficient data from naturally infected individuals for use in systems biological models of analysis, this kind of study is relatively new. The aim of the present study was to investigate the early host response to acute CHIKV infection in adults using a systems biological approach.

We acknowledge that our cohort is a limited representation of the Brazilian population. This relatively low number of subjects is due to emerging nature of CHIKV in Brazil since most research/clinical groups were not fully prepared to collect enough samples and in an adequate manner.

The most clinically relevant symptom related to CHIKV infection is peripheral symmetrical joint pain (primarily inflammatory polyarthralgia). Manifested in several CHIKV cases, it can lead to significant economic impacts and severely affect the patient’s quality of life [12]. The pain starts in the acute phase and can persist for years in up to 50% of the infected adult individuals [51]. Notably, it was proposed that CHIKV infection produced autoimmune sequelae [52]. Although polyarthralgia is rarely detected (5–11%) in children, other kinds of severe manifestations can occur [53]. Since there are neither effective treatments nor licensed vaccines against CHIKV infection, understanding the molecular mechanisms of this complex infection is essential for the development of effective therapies and even vaccines.

The individuals of our cohort were most likely infected with the CHIKV monophyletic group ECSA (East-Central-South Africa) as previously reported [18]. Other lineages can be located although CHIKV is relatively conserved. CHIKV can be genetically classified as West African or Asian, or as belonging to the Indian Ocean (IOL) or ECSA, but the pathogenesis and virulence variation in humans, among these different lineages have not been further investigated [54, 55]. Thus, we believe that in Brazilian regions with different CHIKV strains, the virulence and pathogenesis of the disease may vary.

Consistent with other reports, it was observed that the up-regulation of several genes play a role in the antiviral immune response, and many of them are ISGs [15, 56, 57]. Similarly, other reports that assessed the changes in immune cell subsets during CHIKV infection [50, 58] support the findings of this study regarding the most abundant up-regulated genes being related to neutrophils and myeloid populations and especially dendritic cells and monocytes. The monocytes have been reported recently as important inflammatory and regulatory mediators of the innate immune response to different arboviruses [16, 59]. Corroborating with these findings, the exclusive up-regulation of inflammasome-related genes was also observed. Additionally, the induction of inflammasome activation in macrophages infected with CHIKV *in vitro* was observed. These data explain our previous demonstration that NLRP3 inhibitors can interfere with the pathogenesis of CHIKV virus [30]. Here, we used BMDM from C57BL/6 wild type and Casp1/11^−/−^ mice. In our previous work, we and others showed that the CHIKV infection in wild type mice induced important musculoskeletal diseases and proved it to be an appropriate murine model to study human pathogenesis [30, 60].

Moreover, the negative and significant correlation between CHIKV RNA levels and most eIF family member genes observed here have not been described previously in research regarding CHIKV infections. Furthermore, it is speculated whether the down-regulation of other eIF family members observed in these results in response to the infection could be an important host defense mechanism against the virus replication. Moreover, the protein levels may differ from the gene RNA levels detected in this work. We consider the eukaryotic translation initiation factors as potential markers of the CHIKV arthralgia chronicity and can be better exploited as novel broad-spectrum antiviral targets.

The CHIKV-related arthralgia symptom is significantly similar to rheumatoid arthritis (RA) but there are some important clinical and immunological characteristics that differ between these diseases and the different molecular signatures between both CHIKV and RA can be indicated here. Previous data showed significant concordance between rheumatoid arthritis gene signatures and a mouse model of CHIKV infection/arthritis [14]. Here, we are presenting a more detailed comparison between both diseases in humans. It is well known that the CHIKV-related polyarthralgia affects joints and presents high inflammatory mediators that release locally as a result of the infiltration of mononuclear cells [48, 60]. Therefore, a study comparing the macrophage transcriptome in synovial fluid of both RA and CHIKV infection patients would be highly informative and enriching.

Contrariwise, when compared to the differential gene expressions of DENV infected patients, the data in this study shows specific molecular gene signatures in response to CHIKV. It is interesting to note in this context that although previous infections with ZIKV and DENV were detected in the group of non-CHIKV-infected volunteers, specific responses for CHIKV infection were still identifiable.

Moreover, the comprehensive molecular data in this study showed novel molecules that could play a key role in the acute infection of CHIKV infection and thereby provide new candidates as targets for therapy against this incapacitating disease.

## Methods

### Sample collection and clinical information

Blood samples were collected from subjects reporting arbovirus-like symptoms in the Brazilian states of Sergipe (n = 39 infected and n = 15 controls) and São Paulo (n = 5 controls). Clinical and socio-demographic data were collected through a questionnaire filled out by the patients. Total peripheral blood was collected in Tempus tubes or in heparin-treated tubes that were subsequently transferred to tubes containing RNAlater (Thermo Fisher) and stored at -80°C. A subset of the patients (n = 13) agreed to return for a clinical follow-up. These CHIKV-infected patients were under treatment and their joint manifestations were monitored quarterly. Patients who presented joint pain associated or not with joint or periarticular edema at the onset or significant worsening (in cases of previous joint disease) of the acute febrile syndrome after three months were considered chronic patients.

### Ethics statement

This study was approved by the ethics committees from both the Department of Microbiology of the Institute of Biomedical Sciences at the University of São Paulo and the Federal University of Sergipe (Protocols: 54937216.5.0000.5467 and 54835916.2.0000.5546, respectively). Patient consent was obtained in the written format. All subjects enrolled in this study were adults. Mice experiments and the care of the mice were in compliance with the institutional guidelines on ethics in animal experiments approved by CETEA (Comissão de Ética em Experimentação Animal da Faculdade de Medicina de Ribeirão Preto, approved protocol number 14/2016). CETEA follows the Brazilian national guidelines recommended by CONCEA (Conselho Nacional de Controle em Experimentação Animal).

### Molecular Diagnostics

Real-time RT-PCR was performed to test for CHIKV, ZIKV and DENV as previously described [18, 61]. Nucleic acid extraction was performed using the QIAamp Viral RNA Mini Kit (Qiagen, Valencia, CA, USA) and carried out according to the manufacturer’s instructions. Molecular detection of DENV, CHIKV and ZIKV was performed by using the SuperScript III Platinium One-Step qRT-PCR kit (Invitrogen). We performed the samples’ amplification of the RNAse P in parallel to evaluate the extracted RNA quality. RNA Real-Time RT-PCR reactions consisted of a step of reverse transcription at 50 °C for 15 minutes of the enzyme activation at 95 °C for 2 min, and 45 cycles at 95 °C for 15 s and 60 °C for 1 min for hybridization and extension with the use of ABI 7500 equipment (Applied Biosystems). A cut-off value of Ct (Cycle Threshold) 37 was chosen as the CDC reference assay.

### Serological Diagnostics

Serum samples were evaluated with a commercial semi-quantitative ELISA kit (enzyme-linked immunosorbent assay) that detects anti-CHIKV IgM and anti-CHIKV, Zika and Dengue IgG antibodies. All procedures were carried out according to the manufacturer’s instructions (Euroimmun, Lubeck, Germany). Briefly, sera were diluted in the sample buffer and incubated at 37 °C for 60 min in a microplate well together with a calibrator and the positive and negative controls provided by the manufacturer. The optical density (O.D.) was measured in an Epoch microplate spectrophotometer (BioTek, Vermont, USA) and the results were calculated according to the manufacturer’s instructions. Samples with ratio values (Extinction of the control or patient sample / extinction of calibrator) below 0.8 and above 1.1 were considered negative and positive samples, respectively. Samples with ratio values between 0.8 and 1.1 were considered inconclusive.

### RNA-seq experiments

Total RNA from Tempus tube samples were purified with the Tempus Spin RNA Isolation Kit (Invitrogen). Total RNA from the samples collected in heparin vacutainer tube and maintained in RNAlater were purified using the Ribo Pure blood isolation kit (Invitrogen). Removal of rRNA and globin-encoding mRNA, RNA fragmentation, cDNA generation, adapter ligation and PCR amplification were performed using the TruSeq stranded total RNA with ribo-zero globin sample preparation kit (Illumina). The sequencing of transcriptome libraries was performed on the Illumina HiSeq 1500 platform (Illumina, San Diego, CA). Libraries were prepared using TruSeq Stranded RNA Sample Preparation kit with Poli(A)+ selection quantified through qPCR and sequenced using HiSeq SBS V4 kit (2 x 125 bp paired-end reads).

For RNA-seq preparation, the constructed libraries from 39 infected and 20 control samples were barcoded with dual-indexes of 8bp sequence, multiplexed in 6 technical replicates for each infected sample and 2 technical replicates for each control sample. The prepared libraries were sequenced in two HiSeq Illumina runs (one high-output and another one rapid run mode) where each one of the replicates were sequenced on separated lanes to assess inter-run variability.

The complete set of raw sequences for each assay generated in the HiSeq Illumina 1500 and deposited at the NCBI can be accessed through the BioProject: PRJNA507472 and the BioSample Range SAMN10847030 to SAMN10847088.

### RNA-seq data analyses

Raw paired-end reads were preprocessed for quality control. The Trimmomatic software version 0.36 [62] has been used to remove adapters and contaminants from UniVec database (ftp://ftp.ncbi.nlm.nih.gov/pub/UniVec/), trim the 5′ and 3′ ends with mean quality score below 25 (Phred+33), and discard reads shorter than 40 bp after trimming. We used the parameters “TRAILING:5 HEADCROP:0 LEADING:5 MINLEN:40 ILLUMINACLIP:NextGenPrimerAdaptersUniVec.fa:2:30:10 SLIDINGWINDOW:25:25”. Paired-end reads mapping to PhiX Illumina spike-in were removed using Bowtie 2 version 2.2.5 [63], with the parameter—very-sensitive-local. The processed forward and reverse read files were then paired using Pairfq software (https://github.com/sestaton/Pairfq). After preprocessing, the high quality paired-reads were mapped into the reference genome *Homo sapiens*, version GRCh38.p10 build 38 with the TopHat2 program [64] based on 57,685 genes from *Homo sapiens*, excluding the rRNA and globin genes. For the TopHat2 alignment, we considered the following parameters: minimum intron size (30pb), number of mismatches per read (3pb), number of gaps per read (3pb),—very-sensitive, maximum insertion size deletion (3bp), maximum paired-reads distance (200pb) and only concordant uniquely mapped reads (approximately 92% of the mapped reads) were used for further analyses. To quantify the gene abundance of the mapped paired-end reads in each sample, we used the featureCounts tool from the Bioconductor Rsubread package [65] with the following parameters: GTF.featureType = “exon”, GTF.attrType = “gene_id”, isPairedEnd = TRUE, requireBothEndsMapped = TRUE, minOverlap = value 1, allowMultiOverlap = FALSE, countMultiMappingReads = FALSE. The total number of read counts per gene was obtained from the RNA-seq expression. Normalization of the gene counts was performed with counts per million normalization (CPM), which accounts for differences in library size and adjusts for GC content and gene length. An unsupervised analysis was performed with the R package “PVCA” to estimate the source of variability of experimental effects [66].

The identification of differentially expressed genes (DEGs) was performed comparing each CHIKV patient to all the healthy controls, and following three steps: (1) only genes with counts per million (CPM) > 1 in at least two samples were used in the analyses; (2) differential expression test was carried out using the Bioconductor package edgeR; and (3) DEGs were identified with an adjusted p-value < 0.05 and fold change > 2. The DEGs shared among CHIKV patients were found using the MetaVolcano web tool (https://metavolcano.sysbio.tools/).

The Pearson correlation test (|R| > 0.5 and adjust p-value < 0.01) was used to identify the associations between genes and inverse Ct. R package “randomForest” was used to perform a Random Forest analysis to classify the importance of genes correlated with CHIKV RNA in predicting CHIKV infection.

Regarding the pathways that may be related to the progression of the disease, a Gene Set Enrichment Analysis (GSEA) was performed using as ranks the correlation between the genes and inverse Ct as ranks. A set of Blood Transcriptional Modules (BTM), previously identified by our group [67] through large-scale network integration of publicly available human blood transcriptome, were used as the gene sets.

### Gene co-expression and network analysis

We performed the gene co-expression analysis using the R package CEMiTool [68]. For this analysis, we normalized the expression data using TMM (Trimmed Mean of M-values) and transformed it to a log_2_ scale. We followed the default parameters with a variance filter of 0.2.

To gain a systems-level understanding of the patterns of a certain disease, one of the steps required is the construction and analysis of the network involving the most informative genes. For this, we used the NetworkAnalyst (https://www.networkanalyst.ca/) with the protein-protein interaction (PPI) database based on InnateDB. To improve the visualization, the software Cytoscape (https://cytoscape.org/) and Gephi (https://gephi.org/) were also used.

### Meta-analysis of Dengue and rheumatoid arthritis transcriptome studies

The transcriptome datasets of patients with either rheumatoid arthritis (RA) or Dengue infection were downloaded from the Gene Expression Omnibus (GEO) under the accession codes GSE51808 and GSE94892. DEGs between RA patients and healthy controls and between Dengue-infected patients and non-infected subjects were identified using the limma package (Adjusted P-value < 0.05 and fold-change > 1.25).

### Bone marrow-derived macrophage preparation and infections

Bone marrow-derived macrophages (BMDMs) were prepared using tibia and femur from 6- to 12-weeks-old mice as previously described [69]. Wild type (WT) C57BL/6 mice (Jackson Laboratory, stock number 000664) and Casp1/11^−/−^ [70] derived from C57BL/6 mouse strains were used. All mice were bred and maintained under specific-pathogen-free conditions at the animal facilities of the Medical School Ribeirão Preto (FMRP-USP).

The virus strain CHIKV BzH1 was used and virus stocks were produced by infecting Vero cells (ATCC CCL-81) with a PFU (plaque forming units)/cell of 0.1. Conditioned media used for mock infections was prepared from uninfected Vero cells in a similar manner. The PFU/cell of 5 and the time point of 24 hours of infection was used in most of the *in vitro* experiments using BMDMs, unless otherwise stated in the figure legends.

### Quantification of IL-1β secretion and cell death assay

For *in vitro* cytokine determination and LDH assay, BMDMs were seeded at a density of 2 × 10^5^ cells/well in 48-well plates and pre-stimulated with 500 ng ml^−1^ of LPS (tlrl-peklps; InvivoGen) for 3h, and subsequently infected with CHIKV. The cytokines in the supernatants were measured using a mouse IL-1β ELISA kit (BD Biosciences) according to the manufacturer’s instructions. LDH measurements were performed with CytoTox 96 Non-Radioactive Cytotoxicity Assay (Promega) following the manufacturer’s instructions. The positive control for complete cell lysis and normalization was 9% Triton X-100 (Fisher Scientific); it was incubated with cells for 15 min. The percentage of LDH release was calculated as (mean OD value of sample / mean OD value of Triton X-100 control sample) × 100 and is shown in the figures as the percentage of cell death compared to TritonX-100 (%).

### Caspase-1 evaluation by western blot analysis and endogenous caspase-1 staining using FAM-YVAD–FMK

In order to measure active caspase-1 we used a FLICA assay. Briefly, 10^6^ BMDMs were seeded in 12-well plates overnight, and then infected with CHIKV at a PFU/cell of 5 for 24 hours. As a positive control, we used 20 μM of nigericin (Sigma-Aldrich) for 40–60 minutes. Following this, the cells were harvested and stained for 1 h with a green fluorescent dye that binds specifically to active caspase-1, FAM-YVAD-FMK (Immunochemistry Technologies) following the manufacturer’s instructions. The data were acquired on a FACS ACCURI C6 flow cytometer (BD Biosciences) and analyzed with the FlowJo software (Tree Star).

For the western blot 10^6^ BMDMs were seeded in 6-well plates overnight and subsequently primed with 500 ng ml−1 LPS (InvivoGen, tlrl-peklps) for 3 hours prior to infection with CHIKV. The supernatants were collected, and the precleared supernatants were concentrated thrice in speedvac. They were boiled in a Laemmli buffer, resolved by SDS-PAGE and transferred (Semidry Transfer Cell, Bio-Rad) to a 0.22-μm nitrocellulose membrane (GE Healthcare). The membranes were blocked in a Tris-buffered saline (TBS) with 0.01% Tween-20 and 5% nonfat dry milk. The rat monoclonal antibody to caspase-1 p20 (1:250, Genentech, 4B4) and specific horseradish peroxidase–conjugated antibodies (1:3,000, KPL, 14-16-06 and 14-13-06) were diluted in blocking buffer for the incubations.

Data were plotted and analyzed with the GraphPad Prism 6.0 software (GraphPad, San Diego, California). Multiple groups were compared by two-way analysis of variance (ANOVA) followed by the Bonferroni’s post-test. The differences in values obtained for the two different groups were determined using an unpaired, two-tailed Student’s t-test with a 95% confidence interval. Differences were statistically significant when the p-value was less than 0.05.

## Supporting information

S1 Table Patient information and serological data. (XLSX)

S1 Fig The unsupervised analysis highlights that the variation among individuals can be explained by undefined residual effects. (a) Histogram representing the effects ordered according to their contribution to the sample’s variance measured by PVCA. (b) Unsupervised principal components analysis (PCA) of the 59 subjects, classified according to the expression data. Healthy subjects and CHIKV-infected patients are denoted by the dots colored according to their infection status.
(TIF)

S2 Fig The network of genes whose expression is positively correlated with the levels of CHIKV RNA. Minimum networks constructed using the gene sets that presented a positive NES score with the NetworkAnalyst tool. The red nodes represent genes that are positively correlated with CHIKV RNA, and the color scheme indicates its strength. The gray nodes were added by NetworkAnalyst and are not a part of the correlated genes.
(TIF)

S3 Fig Predictive analysis using machine learning techniques. **(a)** The genes were sorted in the decreasing order of the Predictor Importance of Status used in the random forest model in order to prioritize the genes that most effectively highlight the difference between between infected and healthy individuals. (b) Machine learning parameters of ROC (Receiver Operating Characteristic), sensibility and specificity for 13 different models.
(TIF)

S4 Fig Gene set enrichment analysis of BTM pathways. GSEA was performed for each infected sample against the healthy control to retrieve results from the BTM pathways that were most consistently altered. The BTMs were used as gene sets and their respective log2 fold-change results were considered as rank. Each column represents the results from the GSEA comparison of each infected sample with the healthy control. The pathway names are indicated in the right side of the heatmap. The pathways’ profiles were ordered according to the mean of the NES scores across all patients.
(TIF)

S5 Fig Transcriptome changes in chronic and/or non-chronic subjects compared to healthy individuals. (a) Comparison of the levels of CHIKV RNA (inverse of Ct) between chronic (n = 6) and non-chronic (n = 7) subjects. (b) The number of genes consistently differentially expressed in chronic (brown) and non-chronic (green) subjects. The x-axis represents the number of CHIKV-infected subjects used in each analysis and the y-axis indicates the number of DEGs consistently identified. The green and brown circles represent the number of DEGs in at least 70% of the samples in each group. (c) Representation of DEGs in at least 70% of the chronic and/or non-chronic patients compared to healthy subjects. (d) Genes that overlapped in (c) but that present an inverse behavior depending on the group. (e) Pearson correlation between mean log2 fold-change of chronic patients and non-chronic patients relative to the health controls.
(EPS)
