# Supplementary figures and images for: Systems analysis of subjects acutely infected with the Chikungunya virus
Source: PLoS Pathog. 2019 Jun 18;15(6):e1007880. doi: 10.1371/journal.ppat.1007880 (PMC6599120; doi:10.1371/journal.ppat.1007880)

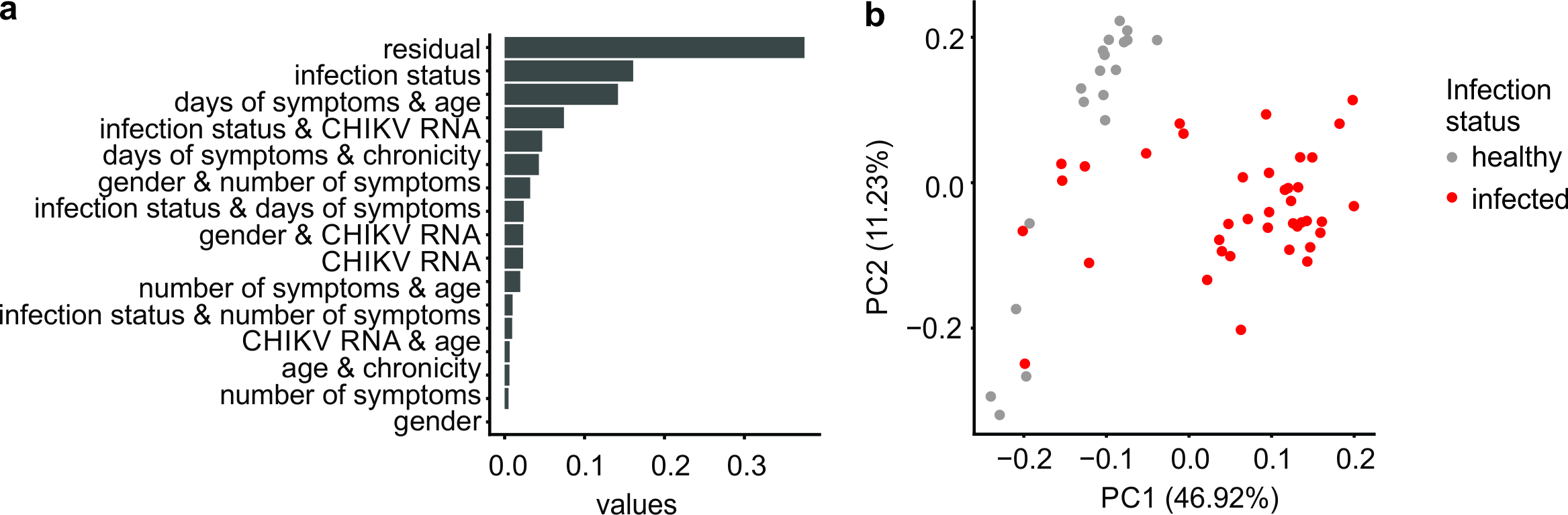

Supplement: S1 Fig — (a) Histogram representing the effects ordered according to their contribution to the sample’s variance measured by PVCA. (b) Unsupervised principal components analysis (PCA) of the 59 subjects, classified according to the expression data. Healthy subjects and CHIKV-infected patients are denoted by the dots colored according to their infection status. (TIF) [file ppat.1007880.s002.tif]

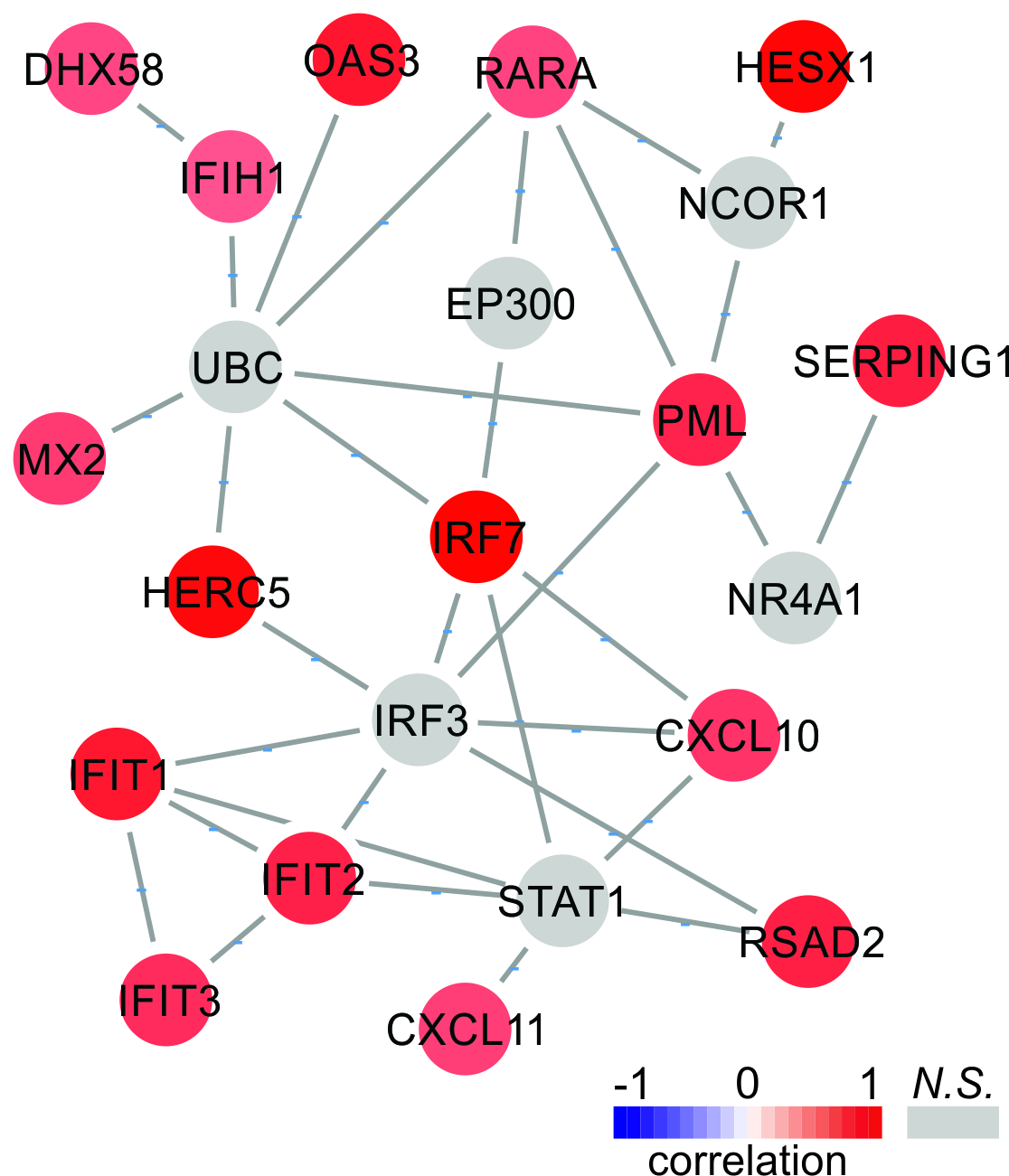

Supplement: S2 Fig — Minimum networks constructed using the gene sets that presented a positive NES score with the NetworkAnalyst tool. The red nodes represent genes that are positively correlated with CHIKV RNA, and the color scheme indicates its strength. The gray nodes were added by NetworkAnalyst and are not a part of the correlated genes. (TIF) [file ppat.1007880.s003.tif]

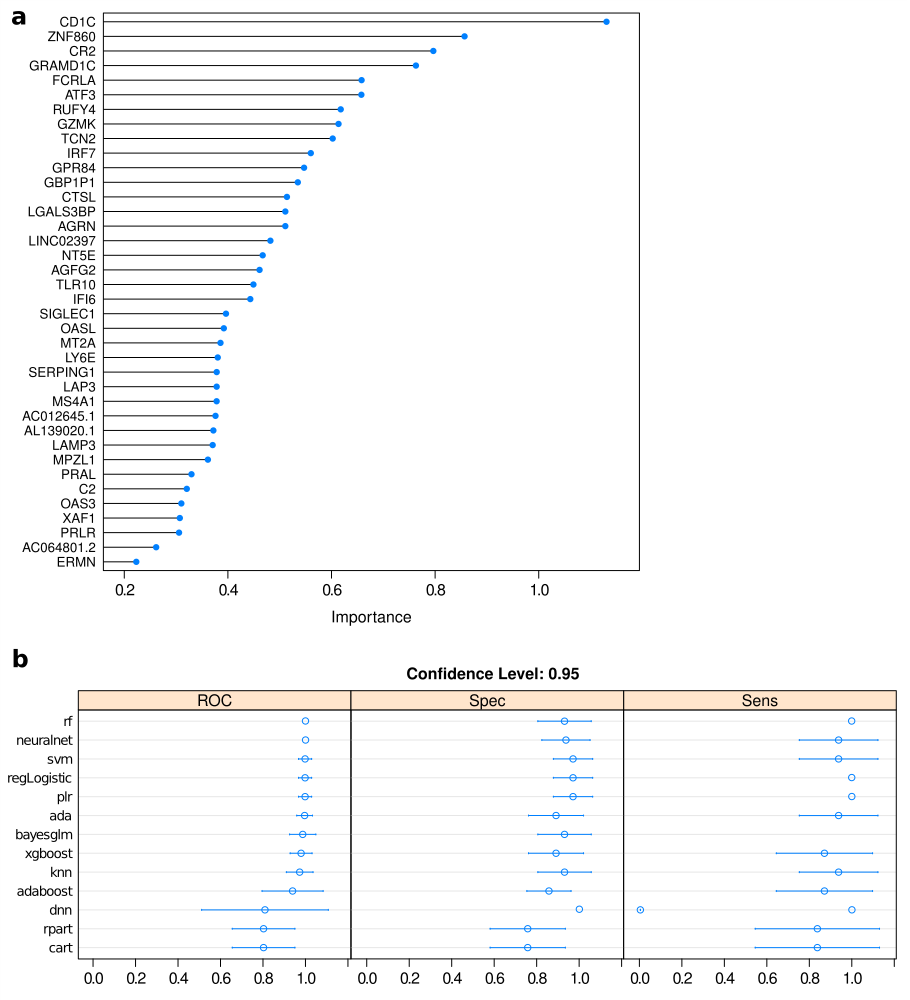

Supplement: S3 Fig — (a) The genes were sorted in the decreasing order of the Predictor Importance of Status used in the random forest model in order to prioritize the genes that most effectively highlight the difference between between infected and healthy individuals. (b) Machine learning parameters of ROC (Receiver Operating Characteristic), sensibility and specificity for 13 different models. (TIF) [file ppat.1007880.s004.tif]

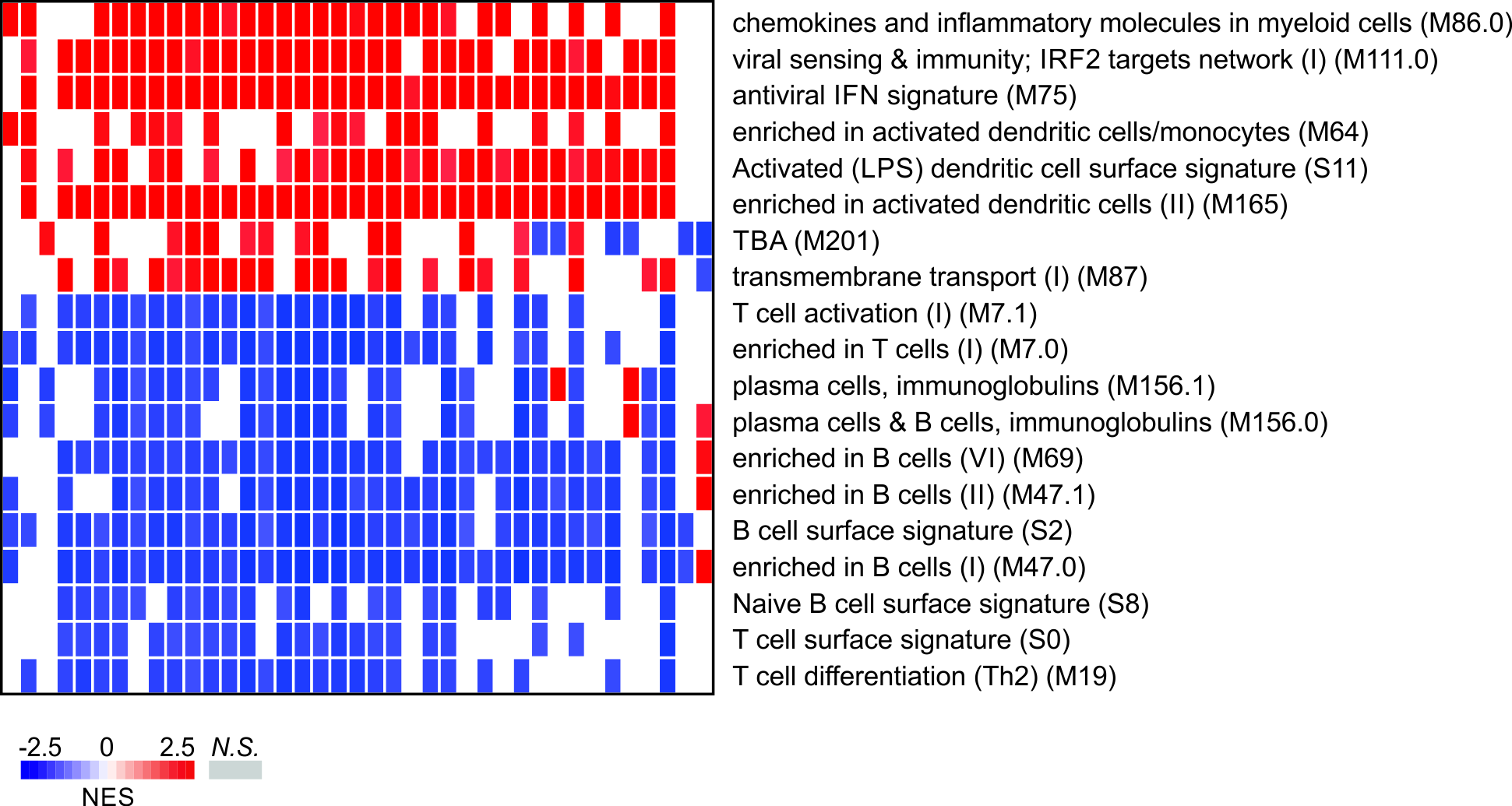

Supplement: S4 Fig — GSEA was performed for each infected sample against the healthy control to retrieve results from the BTM pathways that were most consistently altered. The BTMs were used as gene sets and their respective log2 fold-change results were considered as rank. Each column represents the results from the GSEA comparison of each infected sample with the healthy control. The pathway names are indicated in the right side of the heatmap. The pathways’ profiles were ordered according to the mean of the NES scores across all patients. (TIF) [file ppat.1007880.s005.tif]
